# Numerical simulations of magnetic resonance elastography using finite element analysis with a linear heterogeneous viscoelastic model

**DOI:** 10.1007/s12650-017-0436-4

**Published:** 2017-06-10

**Authors:** Sunao Tomita, Hayato Suzuki, Itsuro Kajiwara, Gen Nakamura, Yu Jiang, Mikio Suga, Takayuki Obata, Shigeru Tadano

**Affiliations:** 1 Division of Human Mechanical Systems and Design, Graduate School of Engineering, Hokkaido University, Kita 13, Nishi 8, Kita-ku, Sapporo, Hokkaido 060-8628 Japan; 2 Department of Mathematics, Faculty of Science, Hokkaido University, Kita 10, Nishi 8, Kita-ku, Sapporo, Hokkaido 060-0810 Japan; 3 Department of Applied Mathematics, Shanghai University of Finance and Economics, 777 GuoDing Road, Shanghai, 200433 People’s Republic of China; 4 Center for Frontier Medical Engineering, Chiba University, 1-33 Yayoicho, Inage -ku, Chiba-shi, Chiba, 263-8522 Japan; 5 National Institute of Radiological Sciences, 4-9-1 Anagawa, Inage-ku, Chiba-shi, Chiba, 263-8555 Japan

**Keywords:** Magnetic resonance elastography, Elastogram, Viscoelasticity, Finite element analysis, Liver

## Abstract

**Abstract:**

Magnetic resonance elastography (MRE) is a technique to identify the viscoelastic moduli of biological tissues by solving the inverse problem from the displacement field of viscoelastic wave propagation in a tissue measured by MRI. Because finite element analysis (FEA) of MRE evaluates not only the viscoelastic model for a tissue but also the efficiency of the inversion algorithm, we developed FEA for MRE using commercial software called ANSYS, the Zener model for displacement field of a wave inside tissue, and an inversion algorithm called the modified integral method. The profile of the simulated displacement field by FEA agrees well with the experimental data measured by MRE for gel phantoms. Similarly, the value of storage modulus (i.e., stiffness) recovered using the modified integral method with the simulation data is consistent with the value given in FEA. Furthermore, applying the suggested FEA to a human liver demonstrates the effectiveness of the present simulation scheme.

**Graphical abstract:**

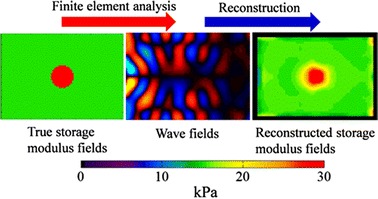

## Introduction

Magnetic resonance elastography (MRE) (Muthupillai et al. 1995, 1996), which consists of magnetic resonance imaging (MRI) and a time harmonic vibration system whose frequency is synchronized with the MRI pulse sequence, is a non-invasive medical diagnostic technique commonly used to diagnose lesions. This system measures the displacement field of a viscoelastic wave in response to an excitation on the surface of the human body. Then solving the inverse problem for a linear viscoelastic partial differential equation (PDE) with respect to the displacement field recovers the storage model of human organs and tissues, which can approximately model the MRE-measured displacement field. Hereafter, we refer to this viscoelastic PDE as the PDE model. This recovery procedure is usually called an inversion, and the stiffness map obtained in the MRE experiment is called an elastogram. Case studies of the inversion algorithm have involved the liver (Huwart et al. 2006; Klatt et al. 2007; Asbach et al. 2010), brain (Klatt et al. 2007; Sack et al. 2008; Green et al. 2008; Zhang et al. 2011), and breast tissues (Sinkus et al. 2005, 2007).

Previous studies have demonstrated the potential of finite element analysis (FEA) to evaluate the inversion algorithm as well as validated the mathematical model of MRE, the recovered storage modulus, and the excitation conditions. Van Houten et al. (1999, 2001) employed a subzone technique algorithm to reconstruct the elastic field by minimizing the difference between the set of measured displacement fields and those computed with FEA (Van Houten et al. 1999, 2001), while Chen et al. (2005, 2006, 2008) used an elastic PDE as a PDE model. Other studies described the damping of waves using subzone MRE techniques employing FEA based on the Rayleigh model (McGarry and Van Houten 2008; Van Houten et al. 2011; Petrov et al. 2014) or the poroelastic model (Perriñez et al. 2009, 2010).

Additionally, FEA has been applied to the viscoelastic equation (Atay et al. 2008; Clayton et al. 2011). Atay et al. demonstrated the reliability of their MRE reconstruction scheme by comparing the reconstructed value to the true value used in FEA, while Clayton et al. computed the viscoelastic waves inside a mouse brain to evaluate the accuracy of one-dimensional (1D) and three-dimensional (3D) inversion algorithms. However, both these studies assumed that the viscoelastic moduli are homogeneous. Ammari proposed optimization-based approach for viscoelastic modulus reconstruction method and the reconstruction method was verified using two-dimensional (2D) heterogeneous FEA (Ammari et al. 2015a, b). Furthermore, to investigate the potential of MRE for the atherosclerotic plaque, Thomas-Seale et al. developed viscoelastic FEA models to compute shear modulus fields of idealized atherosclerotic plaques (Thomas-Seale et al. 2011, 2016). Also, the inversion algorithms were validated based on FEA of cuboids with cylindrical inserts (Hollis et al. 2016a, b). To date, MRE algorithms have yet to be fully evaluated for 3D heterogeneous viscoelastic inversion.

This study aims to evaluate 3D numerical simulations using commercial software called ANSYS (ANSYS Incorporated, 2011) based on the Zener-type PDE model and to test its efficiency using an inversion called the modified integral method (Nakamura et al. 2008; Jiang and Nakamura 2011). We selected commercial software to publicly share the results of FEA for MRE. Additionally, we conducted MRE measurements in agarose gel phantoms with a micro MRI (0.3 T) (Tadano et al. 2012) in Sect. 3.1. A comparison of the simulated results by FEA to the results of the MRE measurements shows that the storage modulus in FEA is recovered with a high accuracy in Sect. 3.2. Furthermore, we applied this inversion to the MRE measurement data of a human liver, and then the measured wave field obtained by FEA of the human liver model was compared with the recovered viscoelastic moduli in Sect. 3.3. Finally, we assessed the accuracy of the FEA model of the liver and the storage modulus recovered from the MRE measurement data. It should be noted that the simulation by FEA, inversion by the modified integral method, and the MRE measurement are linked to each other when evaluating the efficiency of MRE inversion algorithm, and that FEA plays a key role in that link. Furthermore, MRE can be applied to other rheological studies on the viscoelastic properties of soft materials.

## Materials and methods

### Zener-type PDE model

**Fig. 1:**
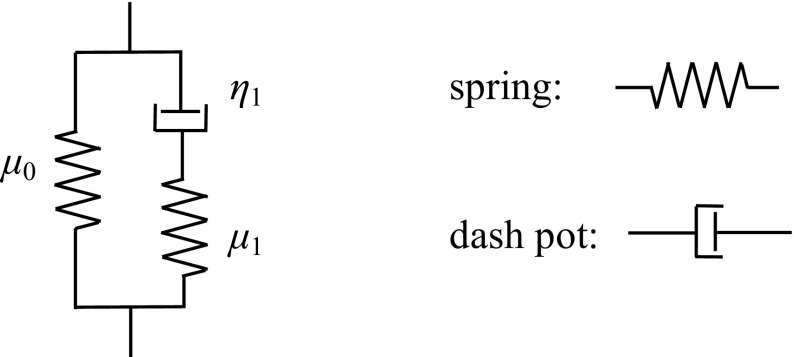
Zener-type model

To obtain the vibration properties of a biological tissue, a linear viscoelastic PDE was used to describe the displacement field of a wave in a tissue (Christensen and Richard 1982). The FEA model based on the Zener model was validated by comparing MRE data to FEA simulated data (Sect. 3.1). Although several viscoelastic PDEs exist, this study used the Zener-type model (three-element Maxwell type model) because its simulated wave images are similar to those obtained from MRE measured data (Jiang and Nakamura 2011). Figure 1 schematically depicts the 1D version of this model, where *μ*_0_ and *μ*_1_ are the spring constants, *η*_1_ is the dashpot viscosity, and *τ*_1_ = *η*_1_/*μ*_1_ is the relaxation time. The stress–strain relation of this model is expressed by:

1 $$\sigma = \int_{0}^{t} {2G} (t - \tau )\frac{{{\text{d}}e}}{{{\text{d}}\tau }}{\text{d}}\tau + I\int_{0}^{t} K (t - \tau )\frac{{{\text{d}}D}}{{{\text{d}}\tau }}{\text{d}}\tau ,$$

where *σ* is the Cauchy stress, *t* is the past time, *e* is the deviatoric strain, *D* is the volumetric strain, and *I* is the identity matrix; *G*(*t*) and *K*(*t*) are the shear and bulk-relaxation moduli, which are described by:

2 $$G(t) = \mu_{0} + \mu_{1} \exp ( - t/\tau_{1} ),$$

3 $$K(t) = \frac{2}{3}\frac{1 + \nu }{1 - 2\nu }\left[ {\mu_{0} + \mu_{1} \exp ( - t/\tau_{1} )} \right],$$

where *ν* is Poisson’s ratio. For the time harmonic vibration, Eq. (1) becomes:

4 $$\sigma = 2(G^{\prime} + iG^{\prime\prime})e\exp (i(\omega t + \delta )) + (K^{\prime} + iK^{\prime\prime})D\exp (i(\omega t + \delta )),$$

where *G*′ and *G*″ are the storage and loss moduli, respectively. *K*′ and *K*″ are the storage and loss bulk moduli, respectively, and *ω* and *δ* are the frequency and phase angle, respectively. The storage modulus and loss modulus are defined by:

5 $$G^{\prime} = \mu_{0} + \frac{{\mu_{1} (\omega \eta_{1} )^{2} }}{{\mu_{1}^{2} + (\omega \eta_{1} )^{2} }}$$

6 $$G^{\prime\prime} = \frac{{\mu_{1}^{2} (\omega \eta {}_{1})}}{{\mu_{1}^{2} + (\omega \eta_{1} )^{2} }}$$

### Modified Stokes equation and modified integral method

Poisson’s ratio *ν* is close to ½ because the tissue is nearly incompressible, which means that *K*′ ≫ *G*′. Asymptotic analysis with respect to the large scaling parameter *K*′*/G*′ indicates that the displacement field *u* can be approximated as a solution for the following boundary value problem (7) or the modified Stokes equation (Jiang and Nakamura 2011; Jiang et al. 2011; Ammari et al. 2007), which is expressed as:

7 $$\left\{ {\begin{array}{*{20}l} {\nabla \cdot [2(G^{\prime} + iG^{\prime\prime})\varepsilon (u)] - \nabla p + \rho \omega^{2} u = 0,} \hfill \\ {\nabla \cdot u = 0,} \hfill \\ {u = f,} \hfill \\ {\partial_{\nu } u: = [2(G^{\prime} + iG^{\prime\prime})\varepsilon (u) - p]\nu = 0,} \hfill \\ \end{array} } \right.$$

where *ρ* is the tissue density, which can be taken as that of water (Fung 1993). *p* denotes the pressure from the longitudinal wave, and *ε* is the linear strain tensor defined by:

8 $$\varepsilon_{ij} (u) = \frac{1}{2}\left( {\frac{{\partial u_{i} }}{{\partial x_{j} }} + \frac{{\partial u_{j} }}{{\partial x_{i} }}} \right) .$$

Furthermore, the first two parts of Eq. (7) are considered in a domain that could be a human body or a phantom Ω. In contrast, the last two parts of Eq. (7) give the mixed type boundary condition with the traction zero boundary condition on part of the boundary of Ω with outer unit normal *n* and the displacement boundary condition with a given displacement *f* on the rest of the boundary where the vibration is given. It should be noted that the displacement field *u* is a complex vector.

If the storage and loss moduli are homogeneous, then applying the curl operator to Eq. (7) removes pressure *p* in Eq. (7). Thus, *w* = ∇ × *u* can be given as:

9 $$(G^{\prime} + iG^{\prime\prime})\Delta w + \rho \omega^{2} w = 0,$$

where Δ denotes the Laplacian. Because applying the curl operator to *u* may amplify the noise, we need to reduce the effect of noise included in *u* (Farahani and Kowsary 2010; Murio 1987). Herein the mollification method (Jiang and Nakamura 2011; Jiang et al. 2011; Ammari et al. 2007) was used for reducing the effect of noise.

Taking the complex conjugate of Eq. (9) gives:

10 $$(G^{\prime} - iG^{\prime\prime})\overline{\Delta w} + \rho \omega^{2} \bar{w} = 0$$

Additionally, integrating the inner product with *w* over a test domain *R* yields:

11 $$G^{\prime} - iG^{\prime\prime} = - \rho \omega^{2} \left( {\frac{{\int_{R} {\left| w \right|^{2} dx} }}{{\int_{R} {w\overline{ \cdot \Delta w} dx} }}} \right),$$

where ‘$\cdot$’ denotes the inner product. Due to the unique continuation property of the solution, the denominator cannot vanish (Jiang and Nakamura 2011). Because the usual algebraic method does not integrate over *R,* $\overline{\Delta w}$ may vanish. This is an advantage of using the formula (11).

Equation (11), which can compute *G*′ − *iG*″ in *R*, is called the modified integral method (Jiang and Nakamura 2011). In particular,

12 $$G^{\prime} = - \rho \omega^{2} \text{Re} \left( {\frac{{\int_{R} {\left| w \right|^{2} dx} }}{{\int_{R} {w \cdot \overline{\Delta w} dx} }}} \right),$$

where Re() denotes the real part in the bracket.

Applying the Zener model (Jiang and Nakamura 2011) the following three important aspects were checked carefully. First, *R* must be at least half of the wavelength. Second, if the heterogeneity of the tissue is sufficiently smooth and the wavelength is sufficiently small, the tissue in a small test domain *R* can be assumed to be homogenous. Finally, even when there is discontinuity in *G*′ − *iG*″, the modified integral equation method still produces satisfactory results.

### MRE experiment with micro MRI

**Fig. 2:**
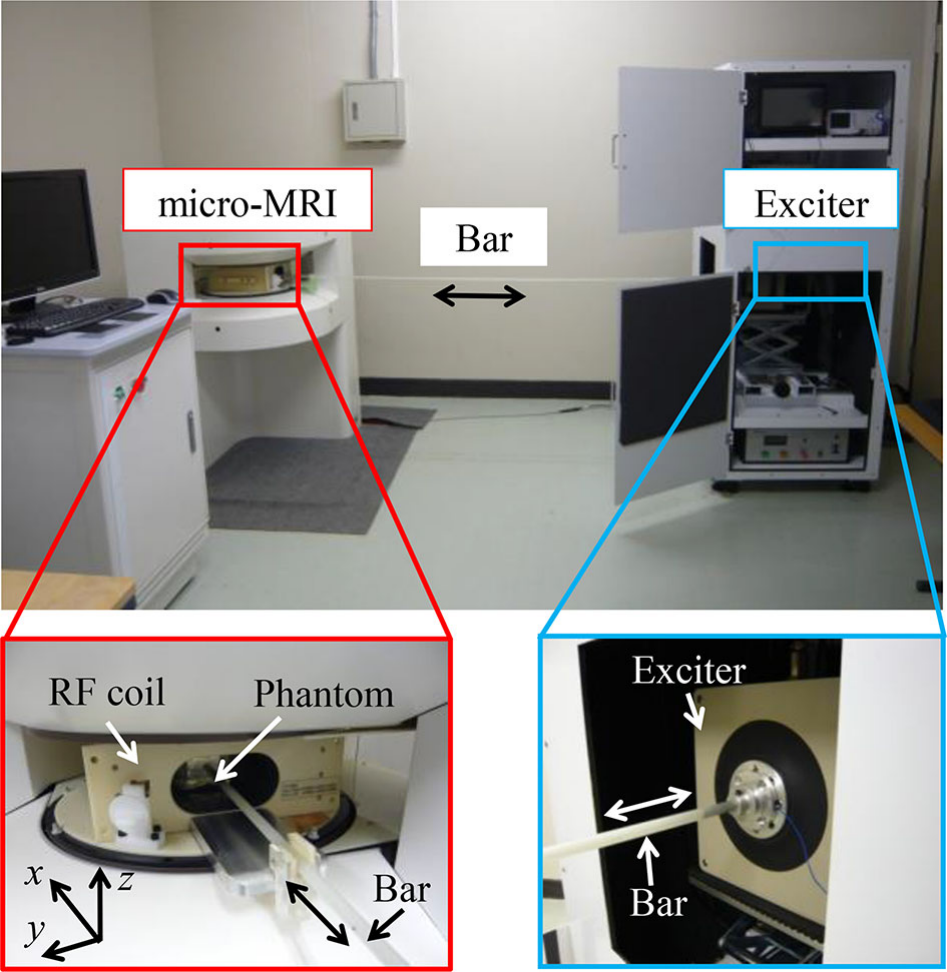
Experimental arrangement for MRE with micro MRI

To validate the numerical simulation, the simulated wave image was compared with the MRE measured wave image obtained by 0.3 T micro MRI (the Compact MRI series, MR Technology, Inc., Tsukuba, Japan). Figure 2 shows the micro MRI. The sample for the MRE measurement was a block agarose gel phantom (100 × 70 × 55 mm) placed in the micro MRI. A longitudinal wave was generated using an electro dynamic generator (C-5010 D-master, Asahi Factory Corp., Tokyo, Japan) as shown in Fig. 2. The wave propagated to the sample through a bar comprised of glass fiber reinforced plastics (GFRP). The diameter of the bar head was 8 mm. The motion of the nuclear spin induced by the local movement of a tissue phantom in a gradient magnetic field induces a phase shift *θ* at position *x,* which is given by:

13 $$\theta (x) = \gamma \int_{{t_{0} }}^{{t_{0} + N/F}} u (t,x) \cdot G(t){\text{d}}t,$$

where *G* is the magnetic field gradient, *F* is the excitation frequency, *u* is displacement fields, γ is the gyromagnetic ratio of characteristic of the nuclear isochromat, and *N* is the number of cycles. Equation (13) can compute the displacement fields *u* from the phase shift *θ*.

### FEA modeling

**Fig. 3:**
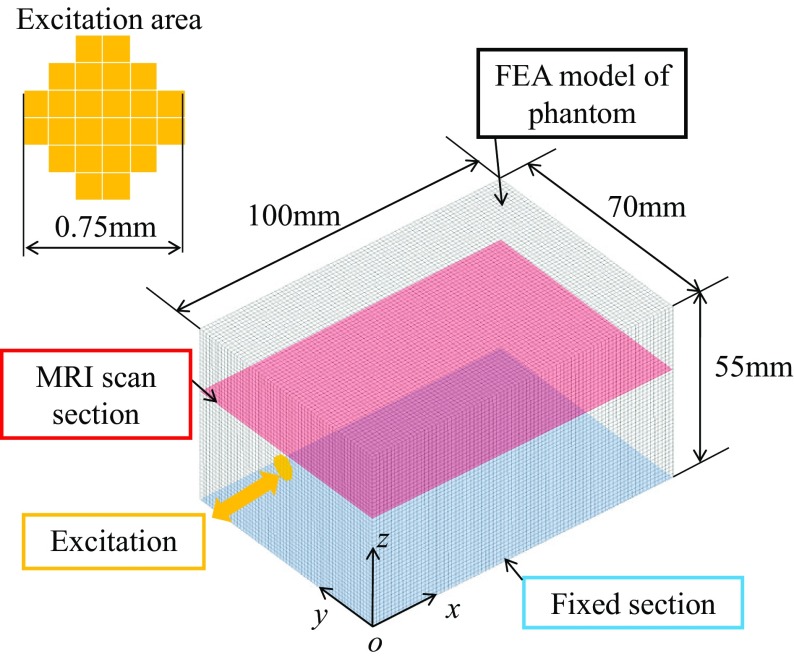
FEA model [100 mm (*x*) × 70 mm (*y*) × 55 mm (*z*)] of the tissue phantom and boundary conditions

Figure 3 shows the FEA model and its boundary conditions. A 3D FEA model of the tissue phantom (100 × 70 × 55 mm) was created and analyzed to obtain its complex displacement fields using ‘harmonic analysis’ in ANSYS (Version 14.0). The steady-state response of the model to sinusoidal excitation was calculated by ‘harmonic analysis’. The model had eight node elements uniformly distributed, and each element measured 1.25 × 1.25 × 1.25 mm. To obtain appropriate wave fields, ten elements per a wavelength are typically necessary. The created FEA model satisfied this condition. All degrees of freedom of the nodes on the bottom surface (an *x*–*y* slice) were fixed (Fig. 3). The nodes included in the center circular 8-mm-diameter region on the *y*–*z* plane were vertically excited in the *x* direction at frequency *f* (=62.5, 125, and 250) [Hz] and 0.5 mm amplitude. The degrees of freedom of the other nodes were not restricted. The storage and loss moduli were computed as their true values of the inverse algorithm using Eqs. (5) and (6) when the mass density *ρ*, Poisson’s ratio *ν*, spring constants *μ*_0_ = *μ*_1_, and relaxation time *τ*_1_ were 1000 kg/m^3^, 0.499 (nearly incompressible), 7.5 kPa, and 0.025 s, respectively. The complex displacement fields *u* in response to the excitation were analyzed with this model and the above conditions.

The original Zener model with the strain–stress relation given by Eq. (1) was used to simulate the displacement field data instead of the modified Stokes model because the Stokes model is an approximation of this Zener model when Poisson’s ratio is very close to 0.5. In this study, this assumption was only used for the inversion. Mollification of the modified integral method was employed for reducing the effect of noise when necessary.

### FEA of the heterogeneous model

**Fig. 4:**
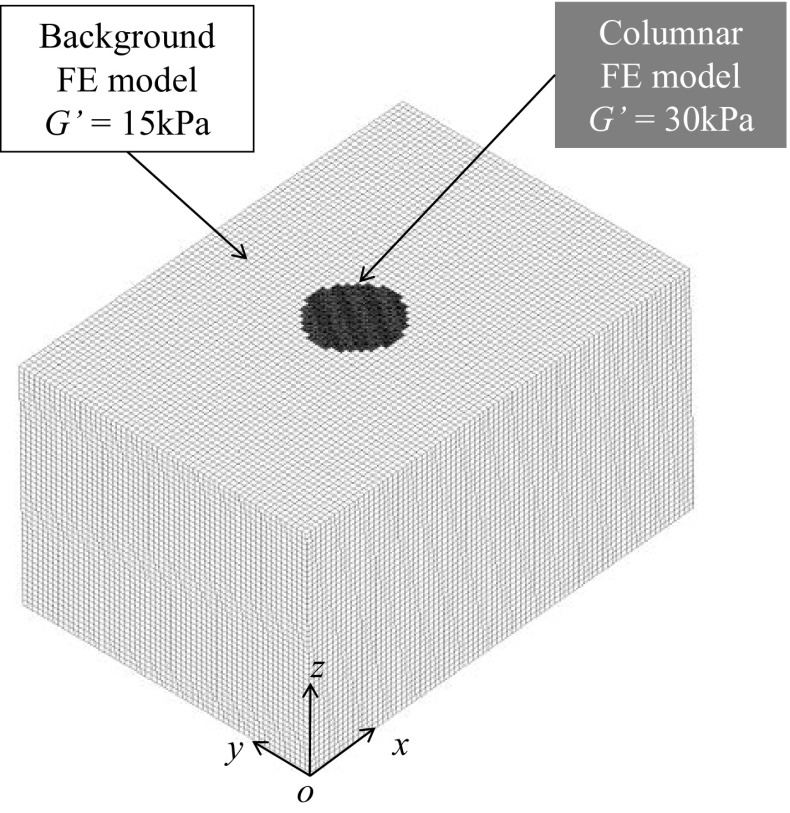
Example of a heterogeneous FEA model of a tissue phantom, including a 20-mm columnar phantom

Next we evaluated the accuracy of the inversion algorithm with respect to the heterogeneous viscoelastic model (Fig. 4). A columnar phantom with a diameter of 10, 15, or 20 mm was embedded in the block model. All columnar phantoms had heights of 55 mm. Eight node elements were used and the element numbers of each columnar phantom were 1056, 2992, and 5456. The viscoelastic parameters of the background gels were the same as those of the homogeneous model. The columnar materials had a spring constant of *μ*_0_ = *μ*_1_ = 15 kPa, while the other parameters were the same as the background parameters. The storage moduli *G*′ of the background and columnar gels were computed from Eq. (5) as 15 and 30 kPa, respectively.

### Validation of the inversion scheme

The inversion method was validated using both homogeneous and heterogeneous models. The viscoelastic moduli were recovered by applying the inversion scheme based on the modified integral method to the displacement field data computed by FEA of the homogeneous model at 62.5, 125, and 250 Hz. Data processing to recover the viscoelastic modulus fields from the FEA results was performed with programs written with MATLAB R2013a (Mathworks). For the inversion, the number of points for the numerical integration in each direction, *N*_*x*_, *N*_*y*_, and *N*_*z*_, was set to 3, and the computations were conducted with the data obtained by FEA (coordinate resolution = 1.25 mm). The numerical integration must have an appropriate number of points for reliable simulations of the MRE inversion scheme. If too few points are used in the numerical integration, a highly accurate storage modulus cannot be recovered. Thus, the parameters must be set appropriately.

To compare the 3D inversion to the commonly used 2D inversion, the 2D inversion method was applied to the computed displacement data on the MRI scan section (*z* = 32.5 mm) in Fig. 3. For the 2D inversion, the number of points for the integral computation was set to *N*_*x*_ = *N*_*y*_ = 3 and *N*_*z*_ = 1. Additionally, the 3D inversion scheme was applied to the heterogeneous FEA results.

### FEA modeling of a human liver

**Fig. 5:**
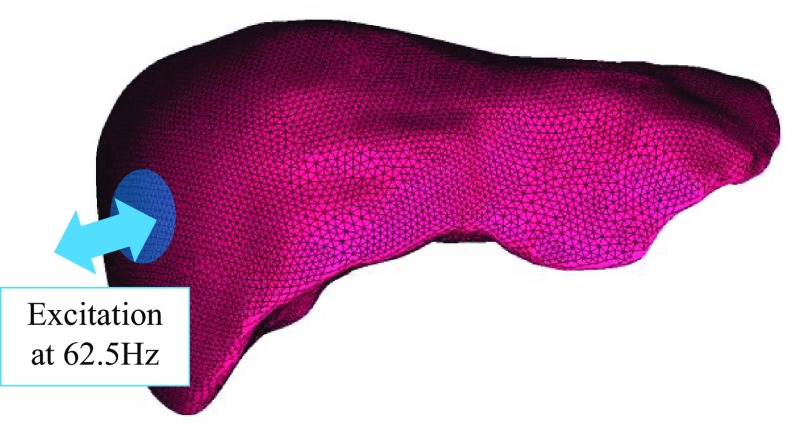
FEA model of a human liver

The 3D FEA of a human liver was conducted to simulate the in vivo MRE experiment of a healthy volunteer (male, age 22 years). The subject provided written informed consent, and the study was approved by the institutional ethics committee in the National Institute of Radiological Science. A spin-echo echo-planar imaging (SE-EPI) pulse sequence with a motion-encoding gradient (MEG) was used to visualize the shear wave pattern in the subject. The experiment was performed with a 3.0 T MRI scanner (Signa HDx; GE Healthcare) using the following parameters: field of view (FOV) = 288 × 288 mm, imaging matrix = 64 × 64, the number of slices = 7, slice thickness = 4.5 mm, TR = 448 ms, and TE = 41.7 ms. To evaluate this experiment by FEA, the 3D shape data of a real human liver extracted from an MRI scanner was used to create the FEA model. The FEA model was discretized via a four-node element with ICEM CFD 14.0. ANSYS 14.0 (ANSYS Incorporated 2011) was employed assuming that the liver model was homogeneous. The interface of the passive drive (diameter 40 mm) section was excited in the normal direction at 62.5 Hz (Fig. 5). The storage modulus was set as the mean of the measured elastogram in the region of interest (ROI) (Fig. 10).

## Results

### Comparison to the experimental image

**Fig. 6:**
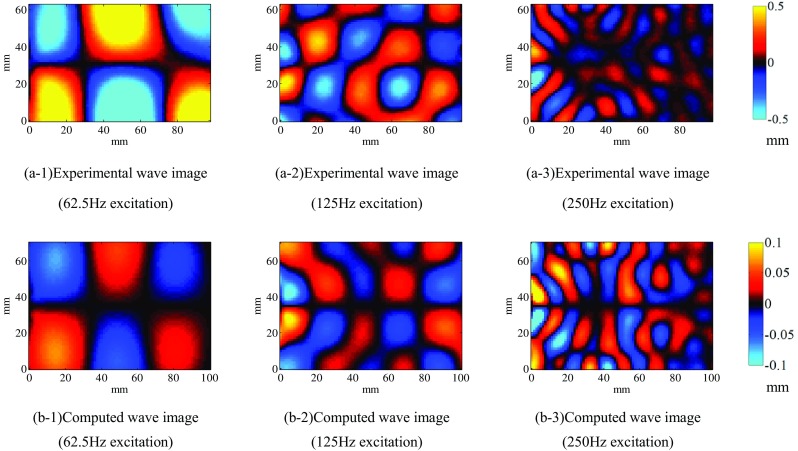
Experimental and computed wave images excited at 62.5, 125, or 250 Hz on an MRI scan slice (*z* = 32.5 mm)

To evaluate the wave propagation model inside a soft tissue, Fig. 6a-1–a-3 shows the MRE displacement field images of 1.2 wt% agarose gel at frequencies of 62.5, 125, and 250 Hz. Figure 6b-1–b-3 shows the 2D displacement fields at *z* = 32.5 mm extracted from the 3D displacement fields computed by FEA. The displacement fields were computed using a homogeneous model and the setup shown in Fig. 3. The spatial wavelength of the displacement field is the dominant parameter for the storage modulus, but the displacement scale is negligible. The experimental and simulated wavenumbers in the images at each frequency (one at 62.5 Hz, two at 125 Hz, and five at 250 Hz) agree, indicating that the spatial wavelengths are qualitatively similar. This finding validates the model employed in this study.

### Validation of the MRE scheme

**Fig. 7:**
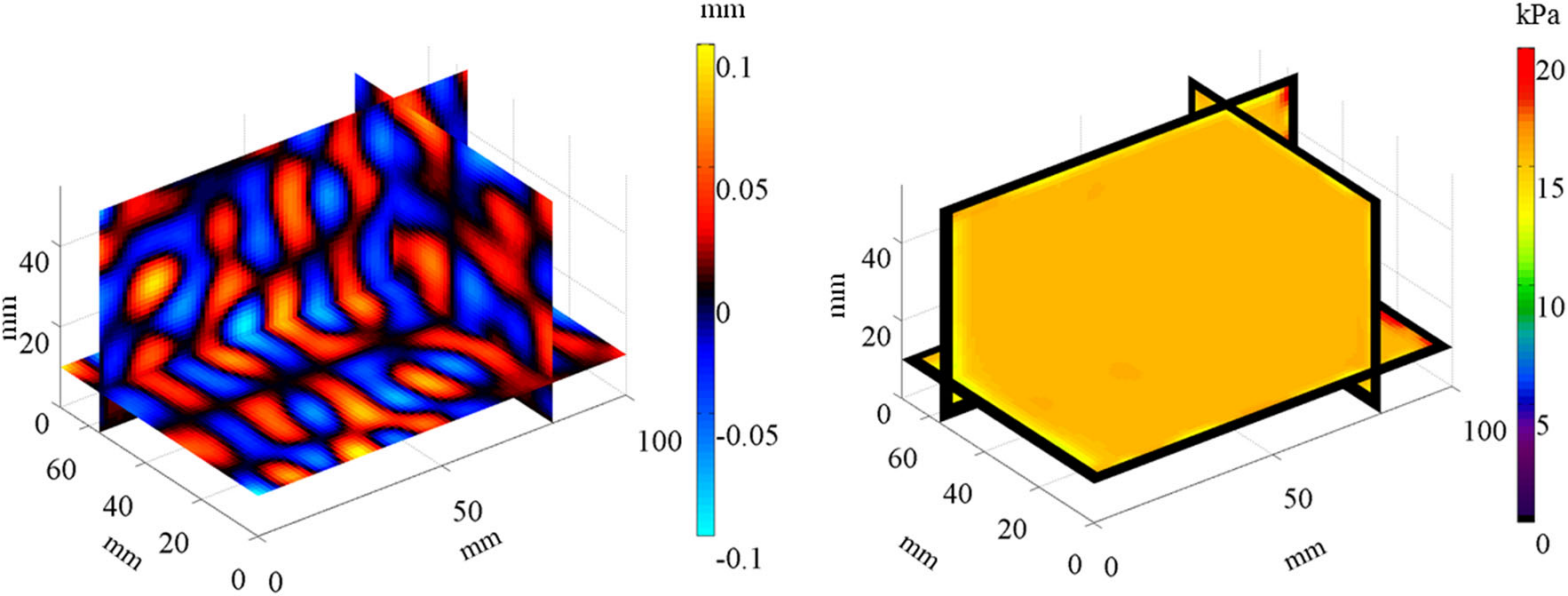
Real parts of the computed displacement fields along the *y* axis and the recovered storage modulus fields (Slice 1: *y* = 57.5 mm, Slice 2: *x* = 81.25 mm, Slice 3: *z* = 11.25 mm) using the same parameters as Fig. 3

**Fig. 8:**
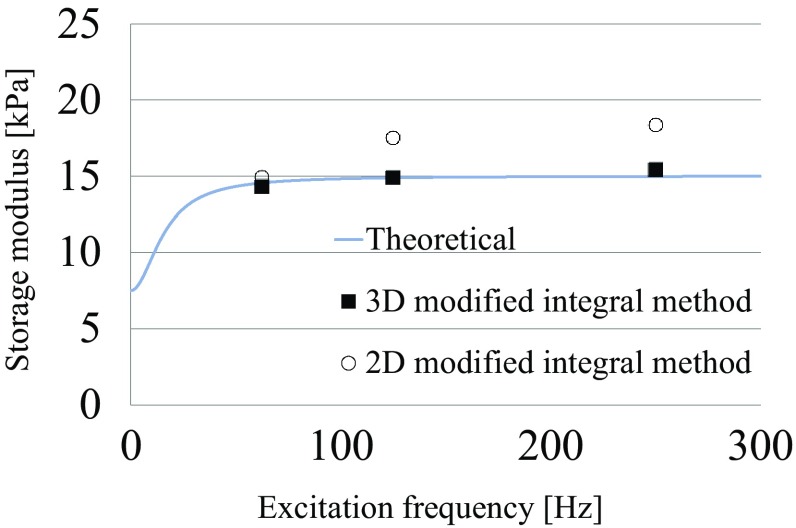
Recovered storage moduli

Figure 7 shows an example of the homogeneous numerical simulation results, where the 3D modified integral method used to numerically simulate the data was applied to the displacement fields in the *y*-direction excited at 250 Hz and the recovered storage modulus fields *G*′. Table 1 shows the averages of the storage moduli recovered from the wave fields excited at each frequency by the inversion scheme. The maximum error between the recovered storage modulus and the measured value is 22.7% in the 2D inversion and 2.7% in the 3D inversion. Figure 8 depicts the relations between the excitation frequency and the recovered storage modulus. The 3D modified integral method reproduces well the theoretical storage moduli at various frequencies.

**Table 1:** Storage modulus recovered from the FEA results

Frequency (Hz)	True (kPa)	3D reconstruction (kPa)	2D reconstruction (kPa)
62.5	14.5	14.3 ± 2.31	14.9 ± 10.3
125	14.9	14.9 ± 0.90	17.5 ± 3.60
250	15.0	15.4 ± 0.75	18.4 ± 3.31

**Fig. 9:**
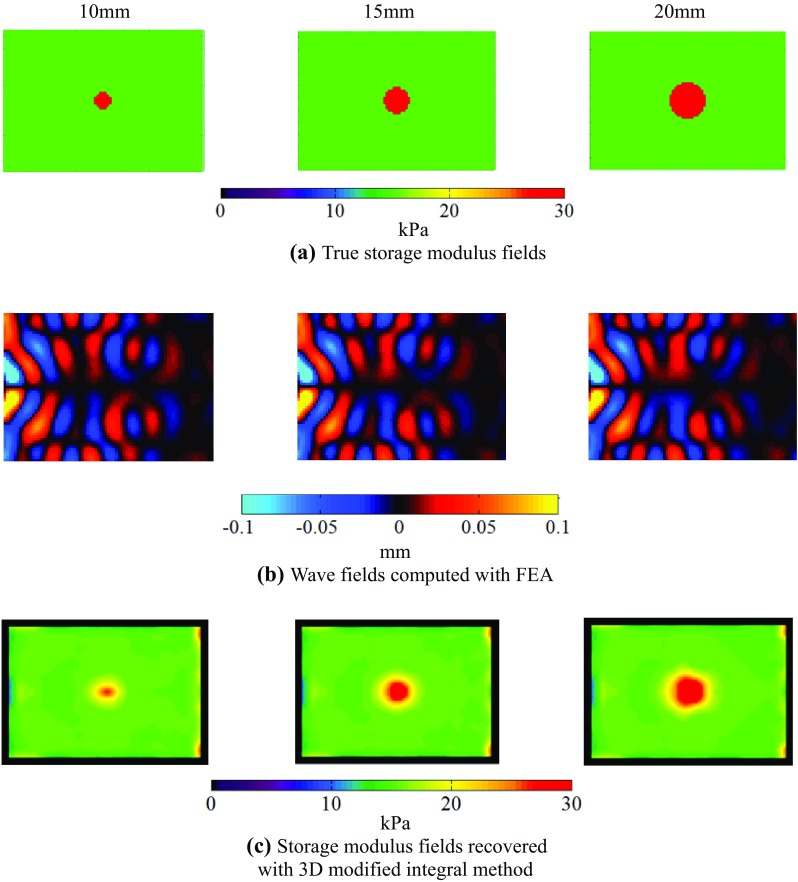
Recovered heterogeneous storage modulus fields at *z* = 32.5 mm

Figure 9a, b shows the storage modulus fields and the computed wave fields in the heterogeneous FEA. Additionally, Fig. 9c depicts the recovered storage modulus fields using the computed displacement fields and the 3D modified integral method. It is observed from Fig. 9 that the modified integral method can reconstruct the heterogeneous elastic modulus fields identifying the difference of the modulus between the base material and the inclusion. These results show that the recovered storage modulus fields and measured values agree well, validating the effectiveness of the inversion method.

### FEA of the human liver model

**Fig. 10:**
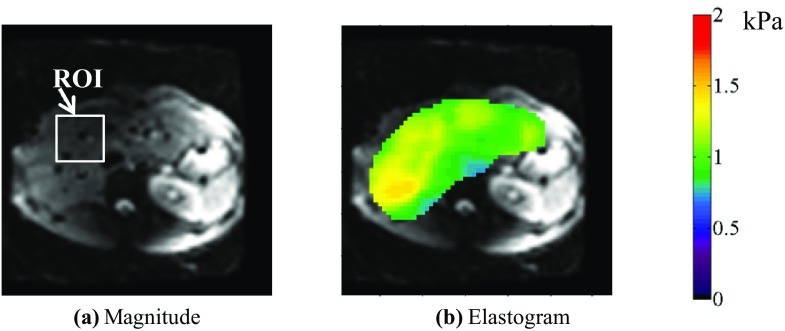
**a** Magnitude and **b** elastogram of the liver in a healthy volunteer

**Fig. 11:**
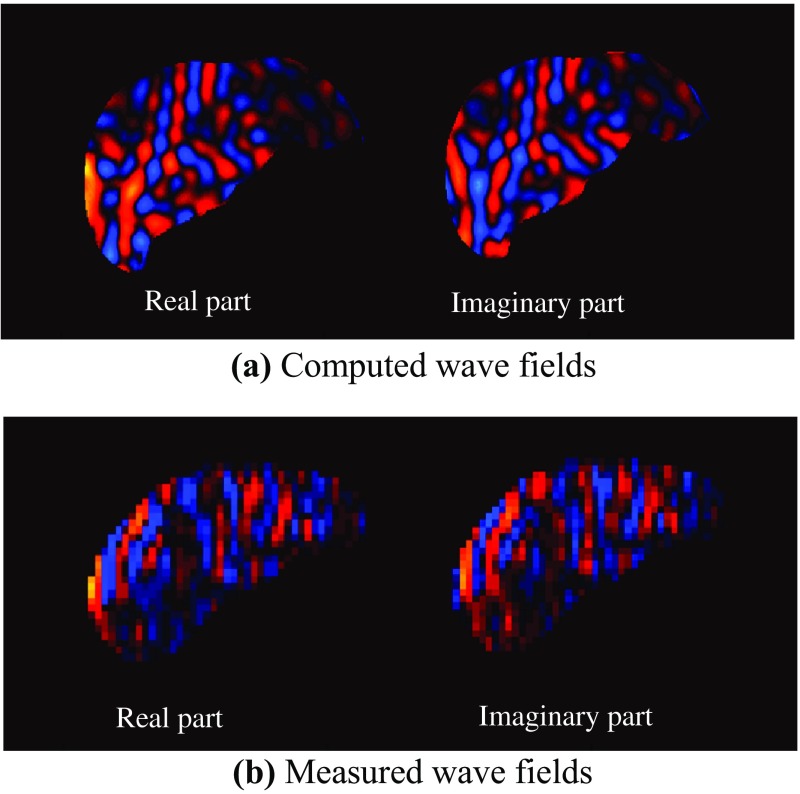
Computed and experimental wave fields propagating from* left* to* right*

Figure 10 shows the magnitude with the ROI and the elastogram of the liver in a healthy volunteer. The mean storage modulus in the ROI is 1.18 kPa. Figure 11 shows the computed wave fields inside a human liver propagating from left to right. To extract the spatial wavelength over the entire region of the liver, the 2D Fourier transforms of the measured wave fields (Fig. 11b) and the FEA wave fields (Fig. 11a) were computed. The 2D Fourier transform yields a predominant peak wavelength of 19.5 mm, while that for the calculated data is 19.2 mm. Because the spatial wavelength of the wave fields corresponds to the storage modulus of the object, the recovered storage modulus is considered to be accurate. This means that the developed FEA can assure the reliability of the reconstruction of viscoelastic modulus by MRE algorithm because FEA was conducted with the reconstructed storage modulus from the measured data.

## Discussion

This study aims to validate the reliability of the MRE measurement by constructing a numerical simulation scheme of the forward problem and the inversion scheme. Initially, to verify the accuracy of the mathematical model used in MRE, FEA of the viscoelastic model was used to simulate the MRE of agarose gel at 62.5-, 125-, and 250-Hz excitations with a micro-MRI system (Fig. 2). A comparison of the MRE wave images of agarose gel phantoms obtained by micro MRI at 62.5-, 125-, and 250-Hz excitations (Fig. 6) shows that the values agree well at all excitation frequencies, confirming that the viscoelastic model set in the forward problem simulation is appropriate to obtain the vibration properties of soft components such as an agarose gel phantom. The slight mismatch is due to difficulties modeling the vibration excitation. Regardless, these results show that the appropriate mathematical model of FEA can be used to simulate wave propagation inside soft tissues.

Next, we verified the accuracy of the inversion method by recovering the storage modulus from the FEA results using the 3D modified integral method (*N*_*x*_ = *N*_*y*_ = *N*_*z*_ = 3). The storage modulus for the FEA simulations is consistent with the recovered storage modulus as there is a less than 3% difference between the two values at all excitation frequencies (Table 1; Fig. 8), demonstrating that the mathematical model of the inverse problem is applicable and inverse analyses are accurate.

The 3D modified integral method was used to recover the storage modulus, and the improvement over the inversion with the 2D displacement fields was evaluated using the 2D displacement fields extracted from the 3D computed displacement fields. A 2D inversion (*N*_*x*_ = *N*_*y*_ = 3, *N*_*z*_ = 1) produces less accurate results because the wavelengths of the 2D displacement images extracted from the 3D displacement images are larger than the true wavelength, which is consistent with previously reported results in multi-slice MRE experiments (Feng et al. 2013). Hence, the 3D inversion measures the storage modulus with a high accuracy.

To simulate MRE for detecting lesions, FEA of the heterogeneous viscoelastic model was conducted. The storage modulus fields recovered with the 3D modified integral method agree well with the measured storage modulus for all sizes of columnar materials (Fig. 9a, b). FEA of MRE is useful to evaluate the elastograms of heterogeneous storage modulus fields, and the 3D modified integral method can recover the heterogeneous storage modulus fields with a high accuracy. Consequently, the 3D modified integral method can determine the storage modulus fields with a high accuracy without changing the wave propagation direction at the boundary section.

In addition, the 3D modified integral method was applied to evaluate the MRE measurement data of a human liver, and FEA of the human liver model was conducted using the geometry of a human liver and the mean of the recovered storage modulus in the ROI. A comparison of the spatial wavelengths of the measured wave fields confirms the effectiveness of FEA for computing wave propagation in a human liver as well as the accuracy of recovered storage modulus (Fig. 11b) and the calculated wave fields (Fig. 11a). Hence, FEA simulations of the MRE can help establish a mathematical model of MRE and assess the accuracy of the inversion. In the diagnosis of liver diseases such as hepatic fibrosis, the recovered storage modulus can be evaluated by comparing with the ideal wave fields computed with FEA.

FEA simulations of MRE can be used to construct a database of various diseases, assuring the reliability of the viscoelastic moduli of human organs estimated using MRE. In addition, applying FEA simulations to the human body geometry can evaluate wave propagation in the human body as well as the excitation conditions (e.g., actuator location, generating power of the actuator, and excitation frequencies for each organ), enabling efficient optimization for each tissue. Thus, the FEA simulations should improve the reliability of medical diagnoses based on MRE.

## Conclusion

FEA simulations of MRE are introduced to evaluate the MRE measurements. FEA of the viscoelastic model can simulate wave propagation inside agarose gel phantom and a human liver. Both homogeneous and heterogeneous FEA simulations validate the accuracy of the inversion method, which was applied to the MRE measurement data of a human liver to evaluate the recovered storage modulus. The 3D modified integral method is highly accurate, demonstrating that FEA simulations are useful to quantitatively evaluate a clinical diagnosis based on MRE.
